# Supplementary material for: Simultaneous readout of multiple FRET pairs using photochromism
Source: Nat Commun. 2021 Mar 31;12:2005. doi: 10.1038/s41467-021-22043-0 (PMC8012603; doi:10.1038/s41467-021-22043-0)
Supplement: Supplementary file 2 — Reporting Summary [file 41467_2021_22043_MOESM2_ESM.pdf]

nature research

Corresponding author(s): Peter Dedecker
Last updated by author(s): Feb 10, 2021

Reporting Summary

Nature Research wishes to improve the reproducibility of the work that we publish. This form provides structure for consistency and transparency in reporting. For further information on Nature Research policies, see our Editorial Policies and the Editorial Policy Checklist.

Statistics

For all statistical analyses, confirm that the following items are present in the figure legend, table legend, main text, or Methods section.

n/a	Confirmed	
☐	☒	The exact sample size (n) for each experimental group/condition, given as a discrete number and unit of measurement
☐	☒	A statement on whether measurements were taken from distinct samples or whether the same sample was measured repeatedly
☐	☒	The statistical test(s) used AND whether they are one- or two-sided<br>*Only common tests should be described solely by name; describe more complex techniques in the Methods section.*
☐	☒	A description of all covariates tested
☐	☒	A description of any assumptions or corrections, such as tests of normality and adjustment for multiple comparisons
☐	☒	A full description of the statistical parameters including central tendency (e.g. means) or other basic estimates (e.g. regression coefficient) AND variation (e.g. standard deviation) or associated estimates of uncertainty (e.g. confidence intervals)
☐	☒	For null hypothesis testing, the test statistic (e.g. F, t, r) with confidence intervals, effect sizes, degrees of freedom and P value noted<br>*Give P values as exact values whenever suitable.*
☒	☐	For Bayesian analysis, information on the choice of priors and Markov chain Monte Carlo settings
☒	☐	For hierarchical and complex designs, identification of the appropriate level for tests and full reporting of outcomes
☒	☐	Estimates of effect sizes (e.g. Cohen's d, Pearson's r), indicating how they were calculated

Our web collection on statistics for biologists contains articles on many of the points above.

Software and code

Policy information about availability of computer code

Data collection	All data collection was performed using the microscope setup described in the methods section of the main article. This setup was controlled using custom software.
Data analysis	- All image processing was performed in IGOR Pro 8 (Wavemetrics). Determination of regions of interest (ROIs), calculation of average intensities and background subtraction was performed using established calculations. These intensity traces were subsequently used for downstream analysis, as detailed fully in the methods sections and/or supplementary information.<br>- Data analysis, calculations and simulations have been performed using custom written code in IGOR Pro 8, and is available at https://bitbucket.org/dedeckerlab/photochromismfret.<br>- All statistical tests were performed in GraphPad Prism 5 (GraphPad Software).<br>- Figures and illustrations were prepared using IGOR Pro 8, Inkscape (v0.92) and Concepts (v2020.12.4).

For manuscripts utilizing custom algorithms or software that are central to the research but not yet described in published literature, software must be made available to editors and reviewers. We strongly encourage code deposition in a community repository (e.g. GitHub). See the Nature Research guidelines for submitting code & software for further information.

Data

Policy information about availability of data

All manuscripts must include a data availability statement. This statement should provide the following information, where applicable:

- Accession codes, unique identifiers, or web links for publicly available datasets
- A list of figures that have associated raw data
- A description of any restrictions on data availability

- Source data are provided with this paper at Zenodo, (https://doi.org/10.5281/zenodo.4392845).

- Addgene plasmids are available for rsAKARev, https://www.addgene.org/166231/, rsAKARev(T/A), https://www.addgene.org/166231/.
- FPBase IDs are available for mTFP0.7, https://www.fpbase.org/protein/2HEJS/; YPET, https://www.fpbase.org/protein/AQACH/; ECFP, https://www.fpbase.org/protein/27HOS/.
- Figures 2, 3, 4, 5, 6 and Supplementary Figures 1, 2, 3, 4, 6, 8, 9, 10, 11, 12, 13 have associated raw data.

Field-specific reporting

Please select the one below that is the best fit for your research. If you are not sure, read the appropriate sections before making your selection.

[x] Life sciences [ ] Behavioural & social sciences [ ] Ecological, evolutionary & environmental sciences

For a reference copy of the document with all sections, see nature.com/documents/nr-reporting-summary-flat.pdf

Life sciences study design

All studies must disclose on these points even when the disclosure is negative.

Sample size	- For each independent experiment, a 35mm glass-bottom dish sample was imaged across 5 different sample positions. For each sample position, multiple regions of interest (ROI) corresponding to single cells were determined.<br>- The number of analyzed cells for each experiment is indicated within the caption of each figure. These sample sizes were chosen so that the each dataset captured the apparent diversity in fluorescence intensities, cell morphologies and light-induced responses across the sample.
Data exclusions	Cells in the field of view with broadly comparable expression and adequate signal of all constructs were considered for selection and downstream analysis. Cells that were too dim, excessively bright, or that appeared unhealthy were excluded. These exclusion criteria were pre-established.
Replication	The number of independent experiments is provided in the figure captions.
Randomization	Samples were allocated into experimental groups based on which biosensors the cells expressed. For the three-biosensor experiment, responses were allocated to three classes based on K-means clustering.
Blinding	All samples were treated equally based on the above-mentioned criteria. Selected cells and corresponding traces were treated by their ROI numbers.

Reporting for specific materials, systems and methods

We require information from authors about some types of materials, experimental systems and methods used in many studies. Here, indicate whether each material, system or method listed is relevant to your study. If you are not sure if a list item applies to your research, read the appropriate section before selecting a response.

Materials & experimental systems

n/a	Involved in the study	
[x]	[ ]	Antibodies
[ ]	[x]	Eukaryotic cell lines
[x]	[ ]	Palaeontology and archaeology
[x]	[ ]	Animals and other organisms
[x]	[ ]	Human research participants
[x]	[ ]	Clinical data
[x]	[ ]	Dual use research of concern

Methods

n/a	Involved in the study	
[x]	[ ]	ChIP-seq
[x]	[ ]	Flow cytometry
[x]	[ ]	MRI-based neuroimaging

Eukaryotic cell lines

Policy information about cell lines

Cell line source(s)	HeLa (ATCC-CCL-2) COS-7 (ATCC-CRL-1651) and HEK293T (ATCC-CRL-3216) were acquired at ATCC, and were regularly replaced from frozen stocks.
Authentication	None.
Mycoplasma contamination	Cell lines were not tested for mycoplasma contamination.
Commonly misidentified lines (See ICLAC register)	No commonly misidentified cell lines were used in the study.
